# Emergent cardiopulmonary bypass during cesarean delivery. A case report

**DOI:** 10.1002/ccr3.3067

**Published:** 2021-05-20

**Authors:** Henrique Vale, James G. Rabalais, Jenna K. Lane, Chawla L. Mason, Arthur L. Calimaran, Daniel Castillo

**Affiliations:** ^1^ University of Mississippi Medical Center Jackson Mississippi USA

**Keywords:** aortic valve, cardiopulmonary bypass, cesarean delivery, infective endocarditis, transesophageal echocardiogram

## Abstract

Successful management of Cesarean Delivery complicated by emergent CPB and AVR requires meticulous multidisciplinary planning. This case also represents the volatility that can arise from severe aortic regurgitation during pregnancy.

## Graphical abstract

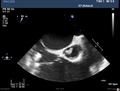

## 1 INTRODUCTION

A 23‐year‐old primigravida at 27.3‐week gestation presented for delivery due to infective endocarditis. A transesophageal echocardiogram demonstrated a large aortic valve vegetation with worsening regurgitation. After induction and delivery, patient went into cardiogenic shock. ACLS and emergent CPB were initiated. Aortic valve replacement was done. Patient was later extubated and discharged.

Infective endocarditis (IE) is rare, with an approximate incidence of 0.006%.^1^ Infective endocarditis in pregnancy is even rarer, affecting 0.001% of all pregnancies.^2^ Medical treatment is an option; however, its effectiveness may be limited. In the event that the patient is nonresponsive to medications, open heart surgery and cardiopulmonary bypass (CPB) during pregnancy or at the time of cesarean delivery (CD) may be unavoidable.^3^ Management guidelines for infective endocarditis in the setting of pregnancy do not exist, and its low incidence limits the ability to conduct randomized controlled trials. Maternal mortality has been reported to be as high as 33%, and fetal mortality can be up to 29%.^4^ Though fetal outcomes can be dismal,^5^, ^6^ there have been a few successes in rescuing both the mother and the fetus.^7^ We present a case of favorable maternal and fetal outcomes improved through successful multidisciplinary collaboration in the management of a parturient who required emergent CPB and aortic valve replacement (AVR) during cesarean delivery. The parturient provided written consent to publish this case report.

## 2 CASE REPORT

A 23‐year‐old primigravida at 20.4‐week gestation with no past medical history presented as a transfer from an outside facility for management of bacterial endocarditis. She initially presented to the outside facility complaining of a 3‐day history of abdominal pain, fever, chills, and back pain that she rated as 10/10 in severity.

**Figure 1:**
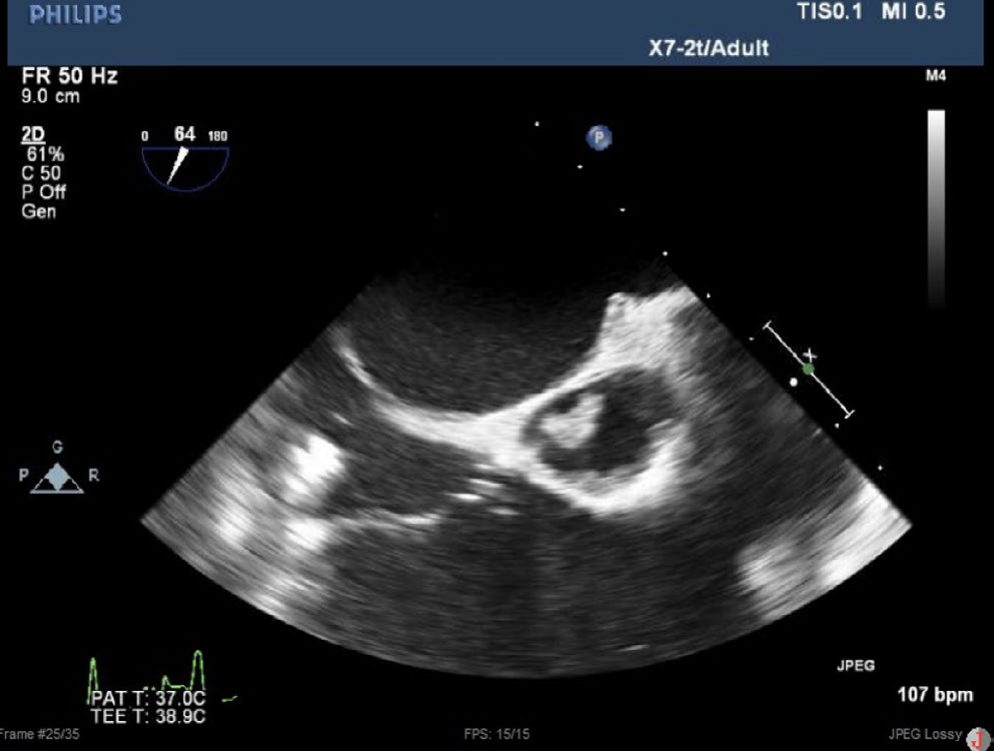
TEE picture of right ventricle inflow outflow tract showing a large aortic valve vegetation in noncoronary cuspid

**Figure 2:**
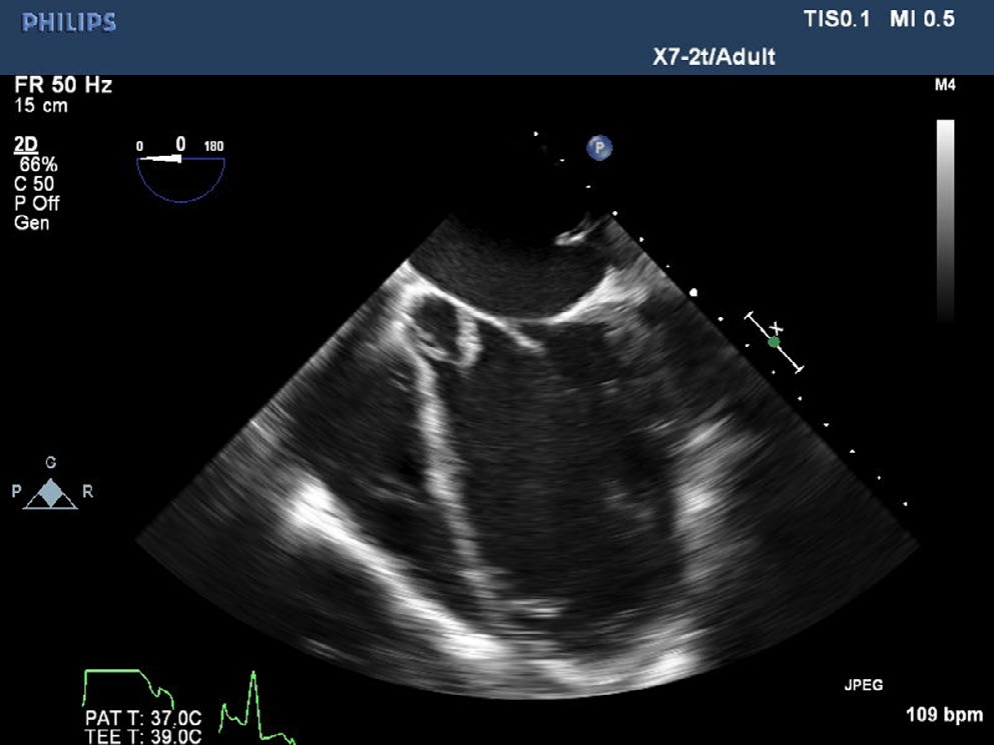
TEE picture of five chamber TEE view showing a large vegetation attached to the noncoronary cuspid of the aortic valve

**Figure 3:**
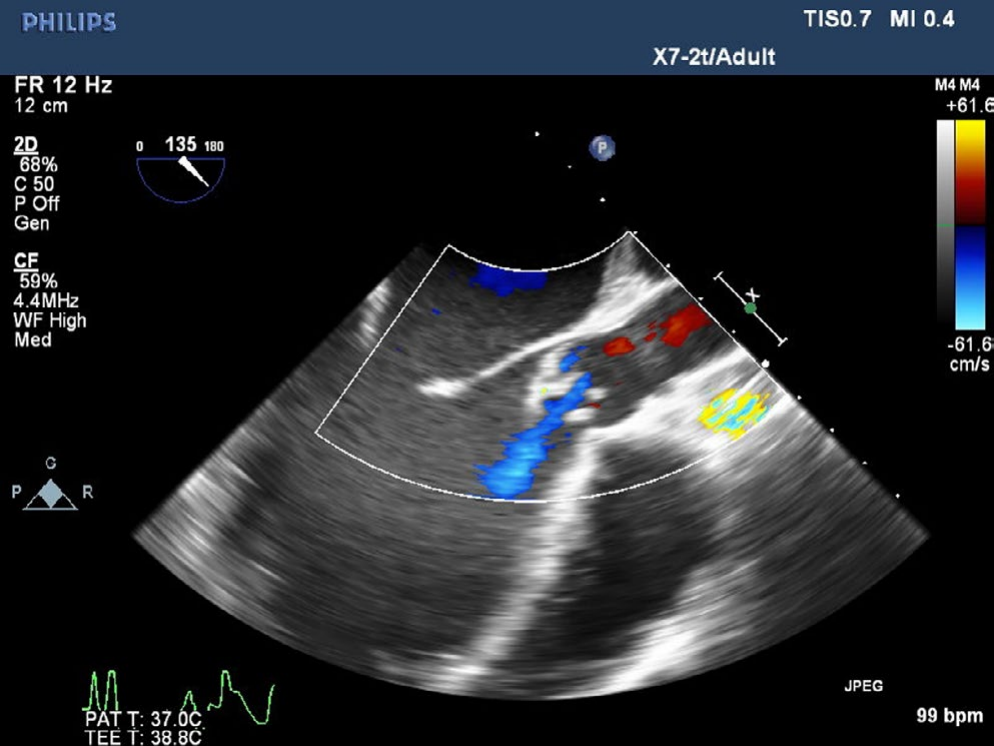
TEE picture of long access view of the aortic valve showing mild aortic insufficiency due to a large vegetation attached to the noncoronary cuspid

On physical examination, temperature was 36.8°C. Cardiac auscultation showed a grade III/VI systolic ejection murmur heard at the right sternal border. Laboratory data indicated a moderate normocytic anemia (hemoglobin 8.0 g/dL) and a vitamin B‐12 deficiency. Blood cultures were positive for methicillin‐sensitive staphylococcus aureus. Transesophageal echocardiogram (TEE) demonstrated a globular, mobile mass on the aortic valve measuring 1.25 × 1.2 centimeters as well as significant aortic regurgitation (see Figures 1, 2, 3, which demonstrate the aortic vegetation). She denied using illicit drugs or a prior history of HIV, hepatitis B, or hepatitis C.

Treatment was initiated with nafcillin IV, based on culture sensitivities, with the plan for patient to receive IV antibiotics for at least 6 weeks. Following 2 weeks of IV antibiotics, the patient was stable and deemed safe for discharge with plan for outpatient antibiotics and follow‐up.

At 24.0‐week gestation, she required readmission due to chest pain and hemoptysis. TEE revealed aortic regurgitation had progressed to severe (see Figures 1, 2, 3). Given the paucity of medical literature to guide this patient's management, several multidisciplinary meetings including cardiothoracic surgeons, infectious disease specialists, obstetricians, cardiologists, maternal‐fetal medicine specialists, neonatologists, and anesthesiologists were held to coordinate the optimal plan of maternal‐fetal care. The patient's symptoms were medically managed while inpatient until 27‐week gestation when the patient complained of worsening dyspnea and TEE demonstrated LV diastolic diameter had increased from 4.9 to 5.6 cm, and diastolic volume had increased from 157 to 197 mL over the past 3 weeks. Collaboratively, the decision was made that fulminant maternal heart failure outweighed the risks of fetal prematurity, and the plan was made to perform elective cesarean delivery in the cardiac OR with cardiothoracic surgeons on standby in case of intraoperative decompensation, with the intent of replacing the native aortic valve 1 week later.

At 27.3‐week gestation, the patient was taken to the OR and induced via rapid sequence induction with cricoid pressure using etomidate and succinylcholine. Delivery of a viable female infant with APGARS 2/7 occurred 3 minutes following tracheal intubation. Immediately after placental delivery, oxygen saturations declined to 59%, and mean arterial pressures markedly decreased from 75 to 27 mm Hg. Chest compressions were initiated, epinephrine 1 mg IV was administered, and the cardiothoracic team proceeded with emergent aortic valve replacement. Cardiopulmonary bypass was initiated 18 minutes after the initial hemodynamic decline with a total ACLS time of 5 minutes.

The durations of CPB and cross‐clamping were 193 and 93 minutes, respectively. An intra‐aortic balloon pump was inserted prior to the discontinuation of CPB. Upon discontinuing cardiopulmonary bypass, the patient was severely vasoplegic and required infusions of epinephrine, norepinephrine, vasopressin and phenylephrine, as well as the administration of methylene blue. The patient required massive transfusion including 12 units PRBCs, 12 units FFP, 20 units cryoprecipitate, 2 pooled platelets, 24 micrograms of desmopressin, and 7 mg factor VII. Upon completion of the procedure, she was transferred to the ICU with a new 21‐millimeter bioprosthetic valve.

Her postoperative period in the ICU was complicated by both cardiogenic and distributive shock. She developed a significant lactemia with acidosis and base deficit despite aggressive volume resuscitation with albumin, blood, and crystalloid fluids. Systemic vascular resistance remained in 400‐500 dyn·s/cm^5^ despite multiple vasopressors. She was noted to be febrile with a maximum temperature of 38.9°C, which was consistent with systemic inflammatory response syndrome (SIRS). Other conditions that required treatment in the ICU included ischemic hepatitis, acute kidney injury, and hypernatremia. Over the next three days, all vasopressors were weaned, and on POD 3, the patient was successfully extubated and the intra‐aortic balloon pump (IABP) was removed. Though she underwent multiple rounds of ACLS and lacked brain perfusion while the cardiothoracic surgeons were cannulating for CPB, the patient did not acquire any neurological deficits or any other postoperative morbidity. She was discharged home on postoperative day 13.

## 3 DISCUSSION

Due to the limited effects of medical treatment alone in treating infective endocarditis, CPB during pregnancy or at the time of cesarean delivery may be unavoidable. CPB can pose significant morbidity and mortality to both the parturient and the fetus, and the limited recommendations and guidelines for this high‐risk procedure pose significant challenges to the anesthesiologist. Reasons to consider early cardiac surgery while the patient is still receiving antibiotic treatment include progressive heart failure (HF) caused by severe aortic or mitral insufficiency, persistent sepsis, and to prevent systemic embolism.^3^ Heart failure is the most frequent complication of infective endocarditis and is subsequently the most common indication for surgery. The symptoms are noted in 42%‐60% of cases of native valve endocarditis, and is more likely to be present when infective endocarditis affects the aortic rather than the mitral valve.^3^ Surgery is not recommended before the first week of antibiotic therapy is completed.^8^ Should it be necessary to perform surgery prior to the patient receiving 1 week of antibiotics, the risk of postoperative valvular dysfunction and relapse will be increased.^9^ Depending on the gestational age of the fetus at diagnosis of infective endocarditis, it may be appropriate to consider terminating a fetus due to the health risks posed to the mother.

Should surgery become necessary in a parturient with infective endocarditis, the decision must be made to either proceed with surgery during pregnancy or deliver the infant at a later date, or to undergo combined cesarean delivery with valvular replacement and CPB. Delaying delivery and proceeding with valve replacement will enable the fetus to mature and potentially decrease morbidity and mortality of prematurity. However, it is well‐recognized that CPB can cause fetal loss due to multifactorial etiologies. First, CPB can cause placental hypoperfusion because uterine blood flow is not autoregulated.^10^ Furthermore, CPB‐related hypothermia can stimulate uterine irritability and initiate contractions which may cause significant hemodynamic changes and potentially result in placental hypoperfusion.^11^ Finally, the gravid uterus can compress the inferior vena cava when the parturient is supine for an extended period of time, leading to placental insufficiency and secondary fetal hypoxia.^10^ Given these risks, the patient must be educated of the potential for fetal death due to CPB.^5^

This parturient valued the life of the fetus above that of her own, and elected to proceed with the delivery and subsequent valve replacement to maximize the safety of the fetus. Combined cesarean delivery and CPB severely increases risk for postpartum hemorrhage due to the anticoagulation required for CPB. This patient experienced postpartum hemorrhage that required oxytocin IV, methylergonovine IM × 2, Hemabate IM × 2, and absorbable Fibrilla to aid in achieving hemostasis. The hemorrhage was significant enough for the surgeons to consider, but ultimately decide against, pursuing cesarean hysterectomy.

It is overwhelmingly likely the patient developed flash pulmonary edema and resultant heart failure due to placental autotransfusion following cesarean delivery. Due to meticulous preparation for this case, cardiac surgeons were present in the operating room and the chest was already prepped and draped to facilitate emergent sternotomy and CPB cannulation. The discontinuation of CPB was delayed due to severe vasoplegic syndrome requiring massive transfusion, likely secondary to the combination of prolonged cardiopulmonary bypass and postpartum hemorrhage in a therapeutically anticoagulated patient.

## 4 CONCLUSION

This case represents the clinical decompensation that can arise from severe aortic regurgitation during pregnancy. Though previous case reports describe successful treatment of infective endocarditis during pregnancy, our case is unique in that the parturient acutely deteriorated intraoperatively and required ACLS with emergent cardiopulmonary bypass cannulation. This case highlights the importance of multidisciplinary approach and Level IV maternal care in achieving optimal maternal‐fetal outcomes.

## CONFLICT OF INTEREST

The authors whose names are listed in this case report certify that they have NO affiliations with or involvement in any organization or entity with any financial interest (such as honoraria; educational grants; participation in speakers’ bureaus; membership, employment, consultancies, stock ownership, or other equity interest; and expert testimony or patent‐licensing arrangements), or nonfinancial interest (such as personal or professional relationships, affiliations, knowledge or beliefs) in the subject matter or materials discussed in this manuscript.

## AUTHOR CONTRIBUTIONS

Henrique Vale, MD: provided patient care during surgery and wrote the manuscript. James Rabalais, MD: provided patient care during surgery and helped writing the manuscript. Jenna Lane, MD: provided patient care during surgery. Chawla L. Mason, MD, FASA: provided patient care during surgery and edited the manuscript. Arthur L. Calimaran, M.D: was part of the multidisciplinary team. Daniel Castillo, MD: was part of the multidisciplinary team. All authors read and approved the final manuscript.
